# CT and MR imaging of primary biliary cholangitis: a pictorial review

**DOI:** 10.1186/s13244-023-01517-3

**Published:** 2023-10-26

**Authors:** Yun Zhang, Tianying Zheng, Zixing Huang, Bin Song

**Affiliations:** 1 Department of Radiology, West China Hospital, Sichuan University, No.37 Guoxue Alley, Wuhou District, Chengdu, 610041 Sichuan China; 2 Department of Radiology, West China Tianfu hospital of Sichuan University, Chengdu, China; 3 Department of Radiology, Sanya People’s Hospital, Sanya, Hainan China

**Keywords:** Primary biliary cholangitis, Computed tomography, Magnetic resonance imaging, Diagnosis, Staging

## Abstract

Primary biliary cholangitis (PBC) is a rare chronic autoimmune-mediated cholestatic liver disease involving medium and small bile ducts that can lead to liver fibrosis and cirrhosis. To date, the pathogenesis of PBC remains elusive, and there is currently no curative medical treatment. Computed tomography (CT) and magnetic resonance (MR) imaging, as common technical tools that allow non-invasive monitoring of liver tissue in vivo, play crucial roles in the diagnosis, staging, and prognosis prediction in PBC by enabling assessment of abnormalities in liver morphology and parenchyma, irregular configuration of bile ducts, lymphadenopathy, portal hypertension, and complications of cirrhosis. Moreover, CT and MRI can be used to monitor the disease progression after treatment of PBC (e.g. the onset of cirrhotic decompensation or HCC) to guide the clinical decisions for liver transplantation. With the optimization of imaging technology, magnetic resonance elastography (MRE) offers additional information on liver stiffness, allows for the identification of early cirrhosis in PBC and provides a basis for predicting prognosis. Gadoxetic acid-enhanced MRI enables the assessment of liver function in patients with PBC. The purpose of this review is to detail and illustrate the definition, pathological basis, and clinical importance of CT and MRI features of PBC to help radiologists and clinicians enhance their understanding of PBC.

**Critical Relevance Statement**

Characteristic CT and MR imaging manifestations of primary biliary cholangitis may reflect the course of the disease and provide information associated with histological grading and altered cellular function.

**Key points**

• Imaging has become highly useful for differentiating PBC from other diseases.

• Key pathological alterations of PBC can be captured by CT and MRI.

• Characteristic manifestations provide information associated with histological grade and cellular function.

• Despite this, the CT or MRI features of PBC are not specific.

**Graphical Abstract:**

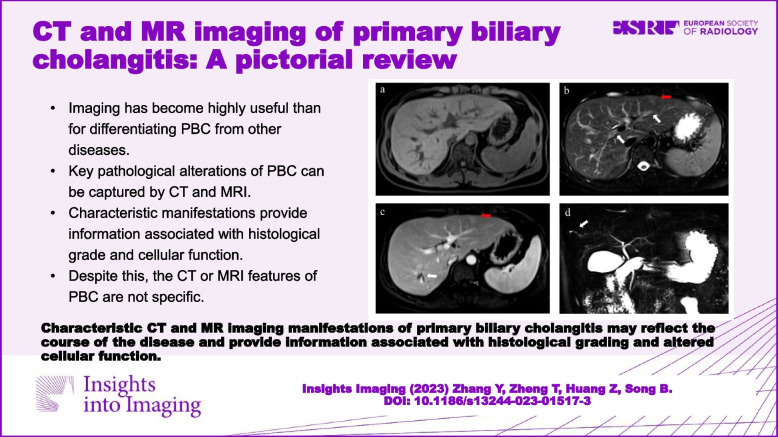

**Supplementary Information:**

The online version contains supplementary material available at 10.1186/s13244-023-01517-3.

## Introduction

Primary biliary cholangitis (PBC) is an uncommon, chronic, autoimmune-mediated cholestatic liver disease. It is characterized by progressive, nonsuppurative destruction of medium to small bile ducts [1]. Although some previous studies have indicated that the development of PBC involves an interaction of multiple factors, including environmental [2–4], genetic/epigenetic [5, 6], and immunological factors [7, 8], the exact origin (or origins) of PBC is still unknown. PBC usually affects middle-aged women and commonly presents as fatigue and pruritus. It can also be asymptomatic, with elevated serum alkaline phosphatase (ALP) or glutamyl transpeptidase (GGT) [9, 10]. The presence of antimitochondrial antibody (AMA) or other PBC-specific anti-nuclear antibodies is highly sensitive and specific for PBC in clinical settings. Ursodeoxycholic acid (UDCA) is recommended as the first-line treatment for PBC. For patients with an insufficient UDCA response, the use of second-line drugs such as obeticholic acid (OCA), fibrates, and budesonide has been shown to be effective in improving patient survival [11]. However, in the absence of proper treatment, there is a high probability of progression to liver fibrosis, leading to end-stage liver disease and a range of complications [12, 13].

In the past, imaging was primarily used for differentiating the disease from extrahepatic biliary obstructive disease. With the update of imaging technologies and the depth of relevant research, our understanding of the imaging manifestations of PBC has gradually improved, and several characteristic imaging features of PBC have been identified that can assist in the diagnosis, staging, outcome monitoring and disease prognosis of PBC. However, previous studies on the imaging of PBC were sporadically reported, lacking a summary, interpretation, and discussion of the clinical significance of the various imaging features of the disease, and did not provide sufficient guidance to radiologists and clinical researchers. The aim of this review is to describe the definition, pathological basis, and clinical importance of the computed tomography (CT) and magnetic resonance imaging (MRI) features of PBC and to summarize the application of the latest MRI techniques in PBC. In addition, the differentiation of PBC from other similar diseases is also briefly discussed.

## Historical background

The designation of PBC, originally known as “primary biliary cirrhosis”, was prompted by the discovery that the formation of xanthoma or jaundice in PBC might be associated with biliary cirrhosis due to inflammatory destruction of small intrahepatic bile ducts [14]. Use of the above nomenclature continued for nearly a decade until it was found that not all patients with PBC develop cirrhosis and that patients have a relatively good prognosis (i.e., the median survival in fatal cases was up to 5.5 years and can exceed 10 years in asymptomatic patients), indicating that “primary biliary cirrhosis” might not accurately reflect the natural history of the disease [15]. It was not until 1965 that researchers discovered that the key serological indicator (i.e. AMA) was associated with the clinical subtype of PBC [16]. This important finding suggested the possibility of diagnosing PBC at an earlier pre-cirrhotic stage and eventually, in 2015, led to a change in the name to “primary biliary cholangitis” [17], which has remained in use to date.

## Epidemiology

PBC is a disease in which female involvement is overwhelmingly dominant (the female-to-male incidence ratio is approximately 3.9–10:1) [1, 18–20]; however, the incidence in male patients has been gradually increasing (female-to-male ratio (3.9–6.2:1) in recent years [21–25]. According to statistics from the 2022 Asia–Pacific Society for the Study of the Liver (APASL) guidelines [11] and the latest meta-analysis, the estimated global incidence and prevalence of PBC are 17.6 and 146 per million, respectively, with North America being the highest, followed by Europe, and the lowest in the Asia–Pacific region. Notably, all three regions showed an upward trend in the incidence and prevalence of PBC, with North America showing the fastest growth in prevalence. In addition, the prevalence of PBC in the Asia–Pacific region has exceeded once deemed and is increasing rapidly. This may represent real growth, or it may be due to the improvement of awareness and ability regarding PBC and its treatment [26]. In addition, with the introduction of UDCA agents, the prognosis of PBC patients dramatically improved, even in those with insufficient response to UDCA [27, 28].

## Histopathology and pathophysiology

**Fig. 1:**
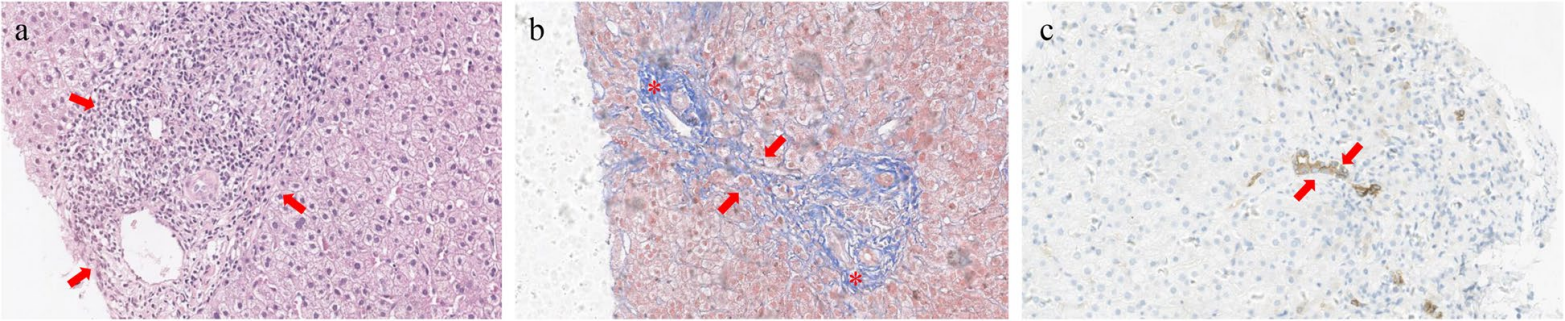
A 53-year-old female with a history of AMA-PBC. **a** Haematoxylin–eosin (HE) staining at 200 × magnification illustrates some infiltrating lymphocytes, monocytes, plasma cells and neutrophils in the portal area (arrows). **b** Masson staining at 200 × magnification reveals fibrous tissue hyperplasia (*), enlargement of the portal tract and bridging fibrosis (arrows). **c** Cytokeratin 7 staining at 200 × magnification highlights biliary metaplasia of hepatocytes (arrows)

The characteristics of PBC are variable and coexist in different stages of the disease, including florid duct lesions, portal tract inflammation, bile duct reduction, fibrosis, and cirrhosis [29] (Fig. 1). Florid duct lesions, defined as nonsuppurative destructive cholangitis, are distinctive in PBC. Parenchymal inflammatory necrosis may be seen but is usually mild. Bile duct reduction is the result of bile duct injury from persistent ductal and portal inflammation, with the smallest bile duct branches being the first to be involved. Portal and periportal collagen deposition finally leads to fibrosis and cirrhosis. However, the premature ductopenic variant, found in less than 10% of patients with PBC, is characterized by sudden and marked bile duct loss that is disproportionate to hepatic fibrosis.

In the multistep pathophysiological process of PBC, the presence of AMAs is key to the autoimmune destruction of biliary epithelial cells (BECs) [30]. The multilineage immune response, including AMAs, targets a specific family of mitochondrial enzymes within BEC, particularly the E2-subunit of the pyruvate dehydrogenase complex (PDC-E2). It also results in the cell death of BECs and eventually the loss of small and medium bile ducts with subsequent cholestasis and fibrosis [8, 30].

## Diagnosis and staging

According to 2022 APASL guidelines, two of the following three conditions must be met to establish the diagnosis of PBC [11]: (1) chronic cholestasis with elevated ALP (other causes of cholestasis are excluded); (2) positive AMA or PBC-specific autoantibodies such as sp100 or gp210 if AMA is negative; and (3) liver biopsy suggesting nonsuppurative destructive cholangitis. However, the presence of atypical clinical phenotypes (e.g. AMA-negative disease) or variants of PBC (e.g. PBC with autoimmune hepatitis [AIH] features) increases the difficulty of diagnosing PBC clinically [11].

The conventional staging systems of PBC include the Ludwig [31] and Scheuer [32] staging systems (Electronic Supplementary Material Table 1), which have long been recommended in guidelines and widely used in PBC research [11]. Recently, some new approaches have been proposed for the staging of PBC. Nakanuma’s staging system showed the strongest prognostic value compared to previous methods, especially in predicting cirrhosis and its complications [33, 34]. Wendum’s staging system showed better correlations with biochemistry than conventional staging systems and exhibited substantial intraobserver and moderate interobserver reproducibility [35]. As mentioned above, liver biopsy is crucial in diagnosing antibody-negative PBC and in disease staging. However, biopsy is invasive and prone to sampling errors. Thus, there is an urgent need for non-invasive markers such as imaging, to provide a complementary role in the diagnosis and staging of PBC when biopsy is not feasible (e.g. when the patient is in poor general condition, in combination with hematological disorders, or unconscious) and in disease follow-up.

## CT and MRI features of PBC

CT and MRI, with their advantages of non-invasive, multiparameter and multiplane imaging, enable visualization of the morphological and functional alterations associated with the development and progression of PBC [36]. The definition, pathological basis and clinical importance of the characteristic CT and MRI features of PBC are summarized in Table 1.

**Table 1:** CT/MRI features of primary biliary cholangitis

**CT/MRI features**	**Definition**	**Pathological basis**	**Clinical significance**	**CT**	**MRI**
**Morphological abnormalities**					
Hepatomegaly [37]	Subjective abnormal diffuse enlargement of the liver supplemented by lineal craniocaudal measurement	Intrahepatic cholestasis, hepatocyte edema, degeneration, massive inflammatory cell infiltration	Found in 11 to 50% of PBCs, the percentage tends to decrease with increasing stage	** + **	** + **
Liver surface nodularity [38, 39]	Nodular liver margin (with nodules < 3 cm)	Diffuse regenerative nodules	Found in 64% of cirrhosis caused by PBC, correlated with advanced stages, used to differentiate stage I-II from stage III-IV fibrosis	** + **	** + **
Liver lobe redistribution [39, 40]	Disproportionate liver lobes due to a combination of segmental atrophy and/or hypertrophy	Altered segmental portal venous perfusion	Segmental atrophy often found in late cirrhosis, less common than viral hepatitis; segmental hypertrophy is common in PBC with stage IV fibrosis	** + **	** + **
**Parenchymal abnormalities**					
Liver parenchymal heterogeneity [38, 41]	Lace-like or patchy density or signal abnormalities in liver parenchyma	Parenchymal necro-inflammatory changes or fibrosis and perfusion changes secondary to destruction of portal vein branches	Degree of heterogeneity on T2WI correlated with fibrosis stages	** − / + **	** + **
Periportal halo sign [42]	0.5–1.0 cm rounded low signal intensity lesion centred on a portal vein branch on T1WI and T2WI	Fibrous tissue deposition or cellular depletion around the portal triads	The most typical feature of PBC, associated with advanced stages	** − **	** + **
Periportal edema [43]	Low-density on CT or hyperintensity on T2WI around portal venous branches	Portal tract inflammation with infiltration of lymphocytes and plasma cells, associated with interfacial hepatitis	Found in 80–100% of early-stage PBC and 66.7% of PBC with stage IV fibrosis, controversial correlation with fibrosis stage	** + **	** + **
**Irregular configuration of the biliary ducts** [43]	Segmental bile duct dilatation, stenosis or poor visualization, mostly involving the intrahepatic secondary bile ducts	Non-suppurative destructive cholangitis	Usually represents more advanced disease, useful for differentiation with PSC	** − / + **	** + **
**Lymphadenopathy** [38, 44]	≥ 2 lymph nodes with ≥ 1 cm on the short axis	Benign reactive hyperplasia of lymph nodes	Found in 62–88% of PBC, located in periportal, gastrohepatic ligament, retroperitoneal, paracardial space and in mesentery, not associated with fibrosis stage or inflammation grade	** + **	** + **
**Portal hypertension**					
Portal vein dilatation [43, 45]	Portal vein diameter > 13 mm	Distortion of hepatic vascular architecture and dynamic changes result in increased resistance to portal blood flow	Can occur in non-cirrhotic stages, associated with histological grade, treatment response and prognosis Splenomegaly may be the first imaging feature captured in PBC, while other signs are more relevant to advanced stages	** + **	** + **
Portosystemic collaterals [43, 45]	Increased number and size of vessels around splenic hilum, paraesophageal region, and gastrohepatic ligament			** − / + **	** + **
Splenomegaly [38, 45]	Craniocaudal diameter of the spleen ≥ 13 cm			** + **	** + **
**Complications of cirrhosis** [11]	Ascites, spontaneous bacterial peritonitis, and HCC development	Portal hypertension and resultant circulation disturbance cause ascites; liver and immune dysfunction cause spontaneous bacterial peritonitis	PBC with baseline cirrhosis is a strong predictor of worse long-term outcomes and should consider liver transplantation once symptoms fail to resolve	** + **	** + **
**Liver stiffness quantification** [46]	Liver stiffness measured by elastography	Hepatic fibrosis and cirrhosis	Used to identify advanced fibrosis of PBC, liver stiffness > 4.30 kPa is a risk factor for cirrhotic decompensation in patients receiving UDCA	** − **	** + **
**Impairment of hepatocyte function** [47, 48]	Semi-quantitative parameters (C (max), T (max) and T (1/2)) and model-free parameters (HEF, irBF, MTT) Mean relative signal enhancement in the liver and mean contrast to noise ratio of the common bile duct	Gadoxetic acid-enhanced MRI visualizes the impairment of hepatocyte function through the uptake and excretion of contrast in the liver parenchyma and bile ducts	Significant differences in several quantitative parameters on gadoxetic acid-enhanced MRI among PBC, postherpetic cirrhosis (other etiologies) and healthy subjects The ability of gadoxetic acid-enhanced MRI in differentiating cirrhosis of different etiologies needs further investigation	** − **	** + **

*CT* computed tomography, *MRI* magnetic resonance imaging, *PBC* primary biliary cholangitis, *T2WI* T2-weighted imaging, *PSC* primary sclerosing cholangitis, *HCC* hepatocellular carcinoma, *UDCA* ursodeoxycholic acid, *HEF* hepatic extraction fraction, *irBF* input-relative blood flow, *MTT* mean transit time

** + **: usually evaluable; − : not usually evaluable; − **/ + **: may or may not be evaluable

### Abnormalities in liver morphology

#### Hepatomegaly

Hepatomegaly is defined as abnormal diffuse enlargement of the liver. Imaging diagnosis of hepatomegaly is mainly based on subjective evaluation supplemented by lineal craniocaudal measurement (e.g. craniocaudal diameter of the liver in midclavicular line of ≥ 15.5 cm) [37, 38]. However, unidimensional measurement cannot accurately reflect liver volume, and a consensus on the threshold of hepatomegaly is still lacking [49]. Hepatomegaly in PBCs is thought to be associated with diffuse intrahepatic cholestasis, hepatocyte edema, degeneration, and massive inflammatory cell infiltration [29]. Approximately 11–50% of patients with PBC present hepatomegaly, with the exact percentage tending to decrease with increasing stage of the disease [37, 38, 44, 50].

#### Liver surface nodularity

The liver margin in patients with PBC can be smooth or nodular (with nodules of < 3 cm) [39]. The configuration of the liver margin is correlated with the size of the underlying regenerative nodules. Gerald et al. [39] reported that 64% of patients with cirrhosis caused by PBC presented with nodular liver margins, and 36% presented with smooth liver margins. However, lobulated liver margins (with more than one nodule > 3 cm) are not seen in PBC. The presence of liver surface nodularity was correlated with advanced stages of the disease and can be used for differentiating stage I–II and stage III–IV fibrosis in PBC[38].

#### Liver lobe redistribution

Lobal redistribution in PBC might be correlated with altered segmental portal venous perfusion, although the exact mechanism is still not fully understood [40]. Segmental atrophy often appears when the disease progresses to late cirrhosis [44]. Atrophy is only seen in 36% of patients with cirrhosis caused by PBC (half with diffuse atrophy and half with segmental atrophy), which is different to that of viral-induced cirrhosis, and focal atrophy of the left medial segment and right anterior segment is uncommon [39]. Previous studies have reported that segmental hypertrophy was common in PBC patients with stage IV fibrosis [37] but less frequent in those with advanced cirrhosis [44]. In PBC-induced cirrhosis, segmental hypertrophy (most common in the left lateral segment and the caudate lobe) is found more frequently than diffuse hypertrophy (56% vs. 12%) [39, 43].

### Abnormalities in liver parenchyma

#### Liver parenchyma heterogeneity

Lace-like or patchy density or signal abnormalities in the liver parenchyma can be seen in PBC, which can be visualized both on unenhanced and contrast-enhanced images. It may be associated with parenchymal necroinflammatory changes or fibrosis and perfusion changes secondary to the destruction of portal vein branches [1, 38]. The degree of heterogeneity on T2-weighted imaging (T2WI) was reported to be significantly correlated with the fibrosis stages of PBC but not with the portal inflammation grade [38, 41].

#### Periportal halo sign

**Fig. 2:**
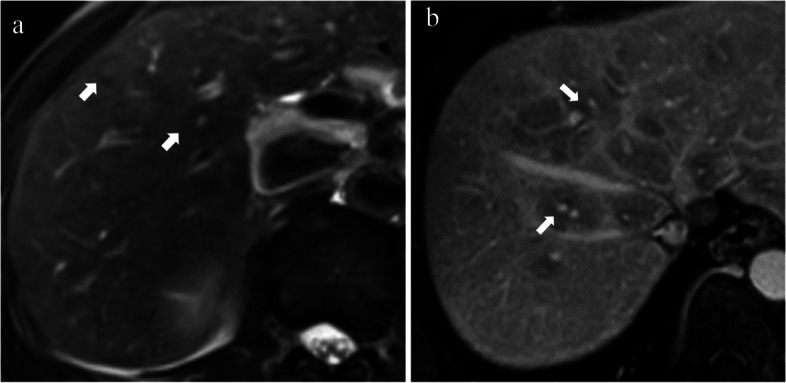
Periportal halo sign of PBC. **a** T2WI image of a 43-year-old female with PBC shows rounded low signal intensity lesions with a diameter of 5 mm to 1 cm centred on a portal vein branch without mass effect (arrows). **b** The portal venous phase (PVP) image of a 53-year-old female with PBC shows rounded low signal intensity lesions without obvious enhancement (arrows)

The portal halo sign is defined as a rounded low signal intensity lesion centred on a portal vein branch with a diameter of 5 mm to 1 cm but no mass effect on both T1-weighted imaging (T1WI) and T2WI and without obvious enhancement (Fig. 2) [42]. This sign may be correlated with the deposition of fibrous tissue and parenchymal extinction around the portal triads. The portal halo sign is the most typical feature of PBC, although it may sometimes also be found in autoimmune hepatitis-PBC overlap [51]. This sign is observed only in patients with later stages of the disease (fibrosis stage II or greater), and the prevalence tends to increase with the increase in the fibrosis stage [37, 38, 52].

#### Periportal edema

**Fig. 3:**
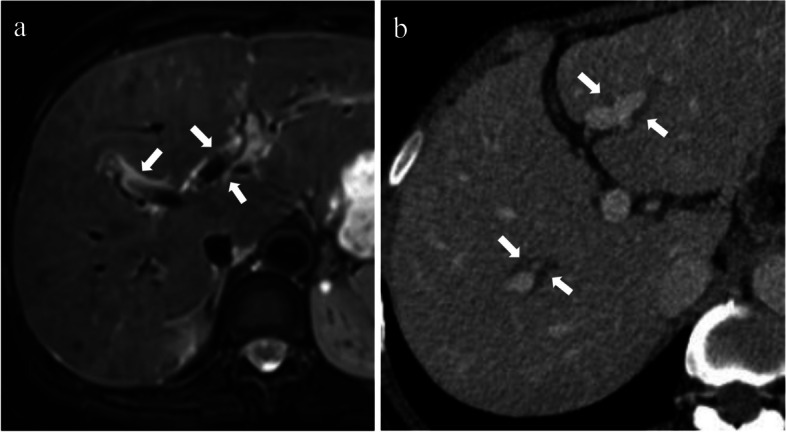
Periportal edema of PBC. **a** T2WI image of a 54-year-old female with PBC shows periportal hyperintensity (arrows). **b** Contrast-enhanced CT of a 53-year-old female with PBC shows periportal low density (arrows)

Periportal edema is not unique to PBC and can be seen in all processes involving ductal hyperplasia, lymphatic dilatation, and inflammatory cell infiltration [43]. This sign has been depicted on MRI in previous studies, mainly presenting as periportal hyperintensity on T2WI [37, 38, 41] (Fig. 3a), but in fact, it can also be seen on CT, where it presents as periportal low density (Fig. 3b). A previous study reported that periportal hyperintensity on T2WI was most common in the early stages of PBC, with a prevalence of 80–100% [45]. As the disease progresses, this sign is less likely to be seen, which is probably because the inflammatory process is gradually replaced by the accumulation of fibrous tissue. However, a recent study demonstrated no significant correlation between periportal hyperintensity on T2WI and fibrosis stage [38]. In addition, Kovač et al. [37] found that approximately 66.7% of patients with PBC with stage IV fibrosis still presented periportal hyperintensity on T2WI, which may indicate that periportal inflammation persists throughout the disease.

### Irregular configuration of the biliary ducts

**Fig. 4:**
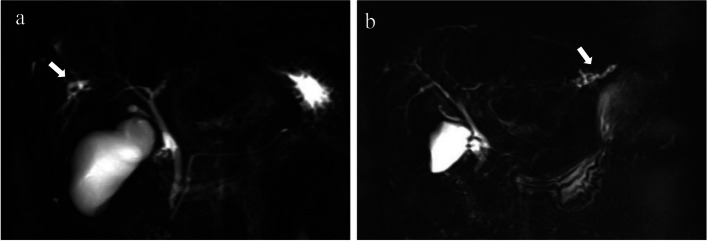
Irregular configuration of the biliary ducts of PBC on MRCP. **a** A 32-year-old female with PBC. MRCP shows irregular dilatation of small bile ducts in the right lobe of the liver (arrow). **b** A 44-year-old female with PBC. MRCP shows segmental dilatation of the small intrahepatic bile ducts in the left lobe of the liver (arrow)

Magnetic resonance cholangiopancreatography (MRCP) may be normal in most patients with PBC, especially in the early stages of the disease when fibrosis in the portal triads has not developed. As the disease progresses to cirrhosis, irregularity and pruning will occur in the intrahepatic bile ducts [43]. Typical bile duct manifestations of PSC, such as beaded dilatation of the intrahepatic and extrahepatic biliary tree, are not seen in PBC. Instead, PBC often presents with segmental small bile duct dilatation, stenosis or poor visualization, mostly with involvement of the intrahepatic secondary bile ducts (Fig. 4).

### Lymphadenopathy

Lymphadenopathy, defined as the presence of more than two enlarged lymph nodes (≥ 1 cm on the short axis), is reported to occur in 62–88% of PBC patients [37, 38, 44]. Enlarged lymph nodes can be seen in the periportal space, gastrohepatic ligament, retroperitoneal space, paracardial space and mesentery [42]. Lymphadenopathy is probably caused by benign reactive hyperplasia and has no obvious correlation with the fibrosis stage or inflammation grade of PBC [38].

### Portal hypertension

Common signs of portal hypertension in PBC include *splenomegaly*, *portal vein dilatation*, *portal vein thrombosis*, and *portosystemic collaterals* [43, 45]. Portal hypertension is common in PBC. However, unlike other chronic liver diseases, portal hypertension can occur in the early stages of PBC before cirrhosis has developed, indicating that portal hypertension is initially presinusoidal [53]. Previous studies have demonstrated that the development and severity of portal hypertension is associated with the histological grade and long-term prognosis of PBC, and changes in the portohepatic gradient can be used to identify responders to UDCA treatment [54, 55]. In addition, splenomegaly is thought to be the first sign that may be captured on imaging in non-hemorrhagic PBC [45]. This may be related to prolonged portal hypertension and possible immune disarrangement in PBC. Other signs, such as the occurrence of portosystemic collaterals, are more relevant to advanced stages of the disease [37].

### Complications of cirrhosis

Patients with advanced PBC can develop all the common complications of cirrhosis, including ascites, spontaneous bacterial peritonitis, variceal bleeding, hepatic encephalopathy, hepatorenal syndrome, and HCC [11]. Among these complications, *ascites*, *spontaneous peritonitis* (e.g. peritoneal thickening and blurring of the abdominal fat planes), and *hepatocellular carcinoma* can be identified and diagnosed on CT and MRI and require the attention of radiologists. PBC with baseline cirrhosis is widely recognized as a strong predictor of worse long-term outcomes, and liver transplantation should be considered once symptoms fail to resolve [11]. Close monitoring of HCC should be emphasized during the follow-up of patients with PBC, especially for high-risk patients with PBC, such as males, patients with advanced-stage disease, and non-responders to UDCA [11]. A recent meta-analysis showed that the pooled HCC incidence in patients with PBC cirrhosis at baseline was 13.05 per 1000 person-years, which was significantly higher than that of PBC patients without cirrhosis (the pooled HCC incidence of 0.35 to 6.02 per 1000 person-years) [56]. Therefore, for PBC patients with comorbid cirrhosis, regular surveillance of imaging by using non-invasive criteria such as LI-RADS allows for earlier detection of HCC and improves patient prognosis [57].

### Liver stiffness quantification

Liver stiffness obtained from ultrasound elastography (USE) has been recognized as a reliable alternative to liver biopsy and histologic diagnosis of fibrosis and cirrhosis [36].

**Fig. 5:**
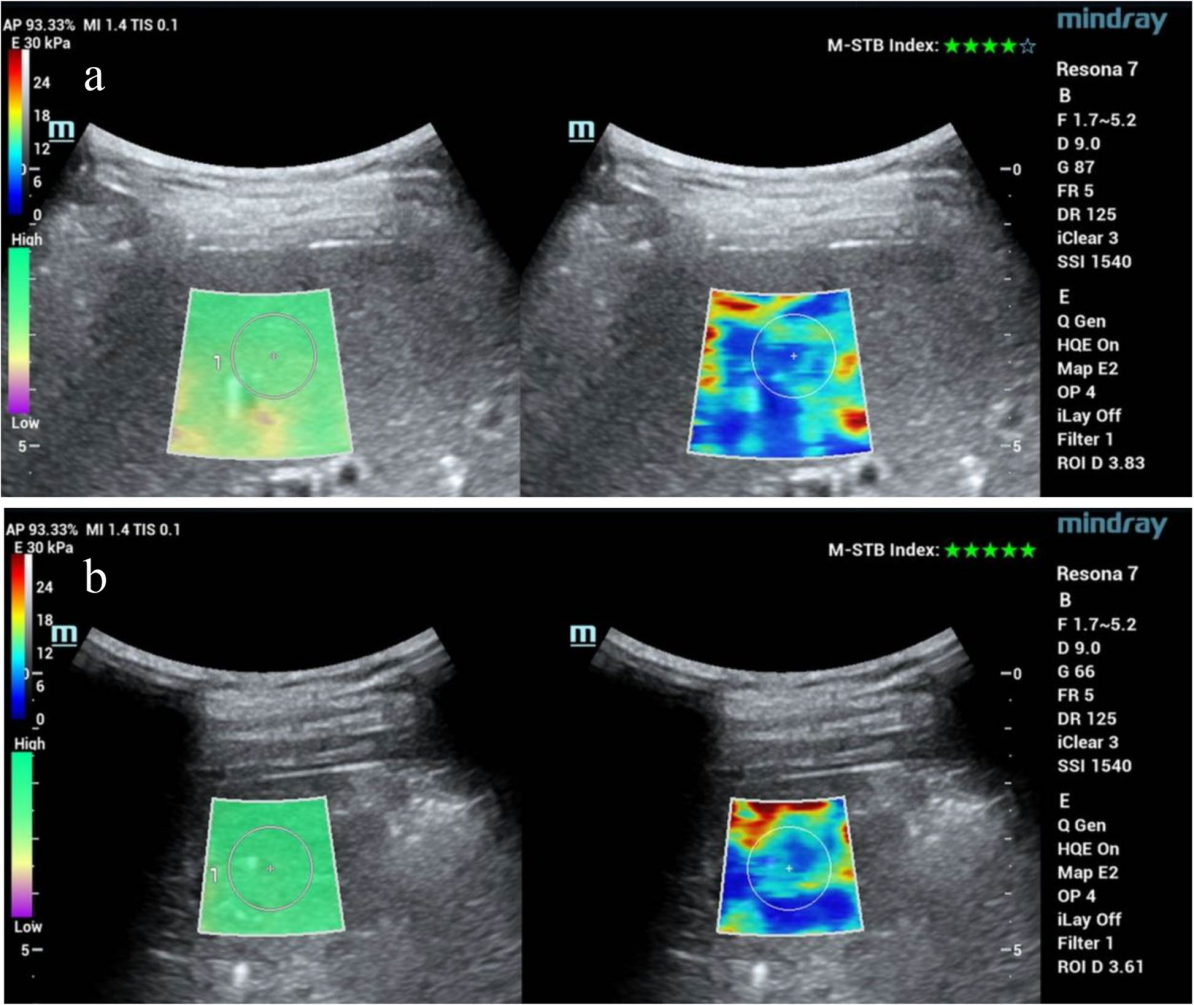
Liver stiffness quantification of PBC on STE. **a** A 62-year-old female with PBC. Liver stiffness measurement was 9.57 kPa, confirmed as PBC stage III (Ludwig staging system). **b** A 27-year-old male with PBC. Liver stiffness measurement was 10.61 kPa, and the patient was diagnosed with cirrhosis. Liver biopsy confirmed as PBC stage IV (Ludwig staging system)

Recently, sound touch elastography (STE) has been introduced to achieve ultra-wide beam tracking imaging. This technique allows all shear waves to be detected within a few hundred microseconds and rapidly calculated, and thus, the results are developed in real time as elastography maps (Fig. 5) [58]. In the study of Luo et al. [59], liver stiffness measurements obtained by STE showed good capability to evaluate liver fibrosis stages in patients with autoimmune liver disease and were superior to the aspartate aminotransferase (AST)-to-platelet ratio index (APRI), and fibrosis-4 (FIB-4) index. In addition, a few published studies [58, 60] indicated that STE performed similarly to other USE technologies, such as shear wave elastography (SWE) and vibration-controlled transient elastography (VCTE) in chronic liver disease (including PBC) differentiation.

Magnetic resonance elastography (MRE) is considered to have equal diagnostic value as ultrasound in providing liver stiffness values and even outperforms ultrasound in monitoring early fibrosis [46, 61]. In PBC, liver stiffness measured by MRE can be used to identify histologically advanced fibrosis and performs better than biochemical indicators. In addition, liver stiffness > 4.30 kPa on MRE was considered to be a risk factor for the development of cirrhotic decompensation in PBC patients treated with UDCA [62].

### Impairment of hepatocyte function

Hepatobiliary contrast agents, i.e. gadoxetate disodium (Gd-BOPTA) and gadobenate dimeglumine (Gd-EOB-DTPA), are increasingly being used for MRI-based diagnosis of cirrhosis or HCC [63]. The results of Liu et al. [64] have shown that the biliary imaging derived from the hepatobiliary phase of Gd-BOPTA-enhanced MRI could be used as a valuable tool in the prediction of hepatic decompensation and insufficiency for cirrhotic patients. However, Gd-EOB-DTPA has a pharmacokinetic profile different from that of Gd-BOPTA. In cirrhotic patients, as reported, Gd-BOPTA-enhanced MRI yielded lower enhancement of the hepatic parenchyma and lower contrast of the portal vein than Gd-EOB-DTPA-enhanced MRI in the portal venous phase [65]. Given this, the hepatic parenchyma from the hepatobiliary phase with Gd-EOB-DTPA-enhanced MRI of cirrhotic patients demonstrates better compared to Gd-BOPTA-enhanced MRI [66].

Previous studies have revealed important differences in several quantitative parameters related to hepatic blood perfusion on Gd-EOB-DTPA-enhanced MRI between patients with PBC and healthy subjects [47]. In addition, the mean relative signal enhancement in the liver of PBC patients at 4, 20, and 50 min and the mean contrast-to-noise ratio of the common bile duct were significantly different compared with all post-hepatitic cirrhosis patients of the same Child‒Pugh classification. However, the ability of hepatobiliary phase imaging to differentiate different etiologies of liver cirrhosis (i.e. PBC or viral hepatitis) needs further investigation [48]. In addition, some new scoring methods derived from Gd-EOB-DTPA-enhanced MRI such as the functional liver imaging score (FLIS), may serve as a reference for future studies of PBC [67].

**Fig. 6:**
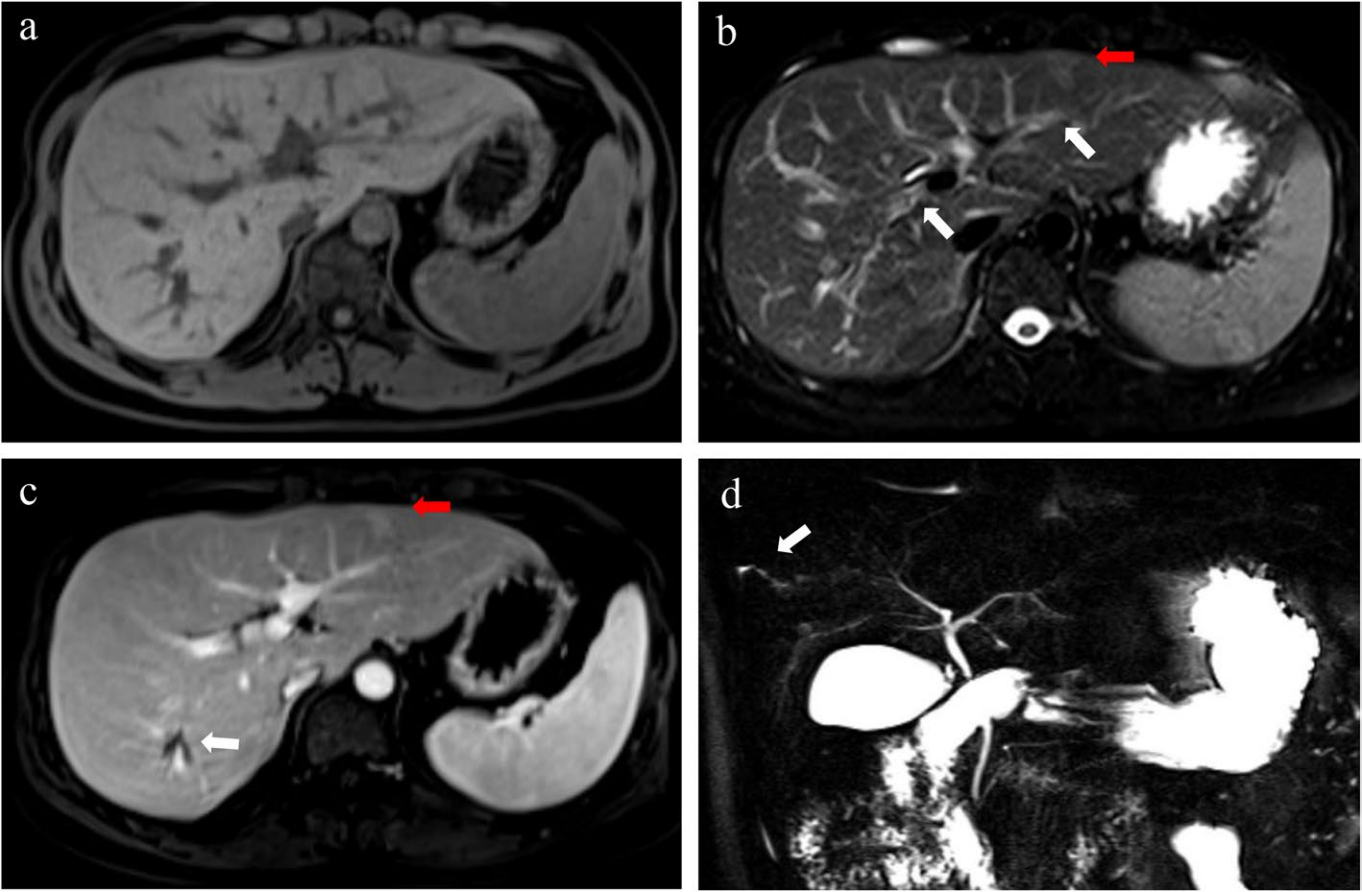
Contrast-enhanced axial MRI scans in a 25-year-old female with PBC (stage I–II, Ludwig staging system) (**a**–**d**). The liver has a regular contour without nodular changes of the surface on the T1WI image (**a**). The T2WI image shows periportal hyperintensity (white arrows), and small patchy heterogeneity in segment II (red arrow) (**b**). The PVP image shows mild heterogeneous enhancement of the liver parenchyma (red arrow) with dilated small bile ducts in segment VII (white arrow) (**c**). The MRCP image shows segmental irregular dilatation of the small bile ducts in the right lobe of the liver (arrow) (**d**)

**Fig. 7:**
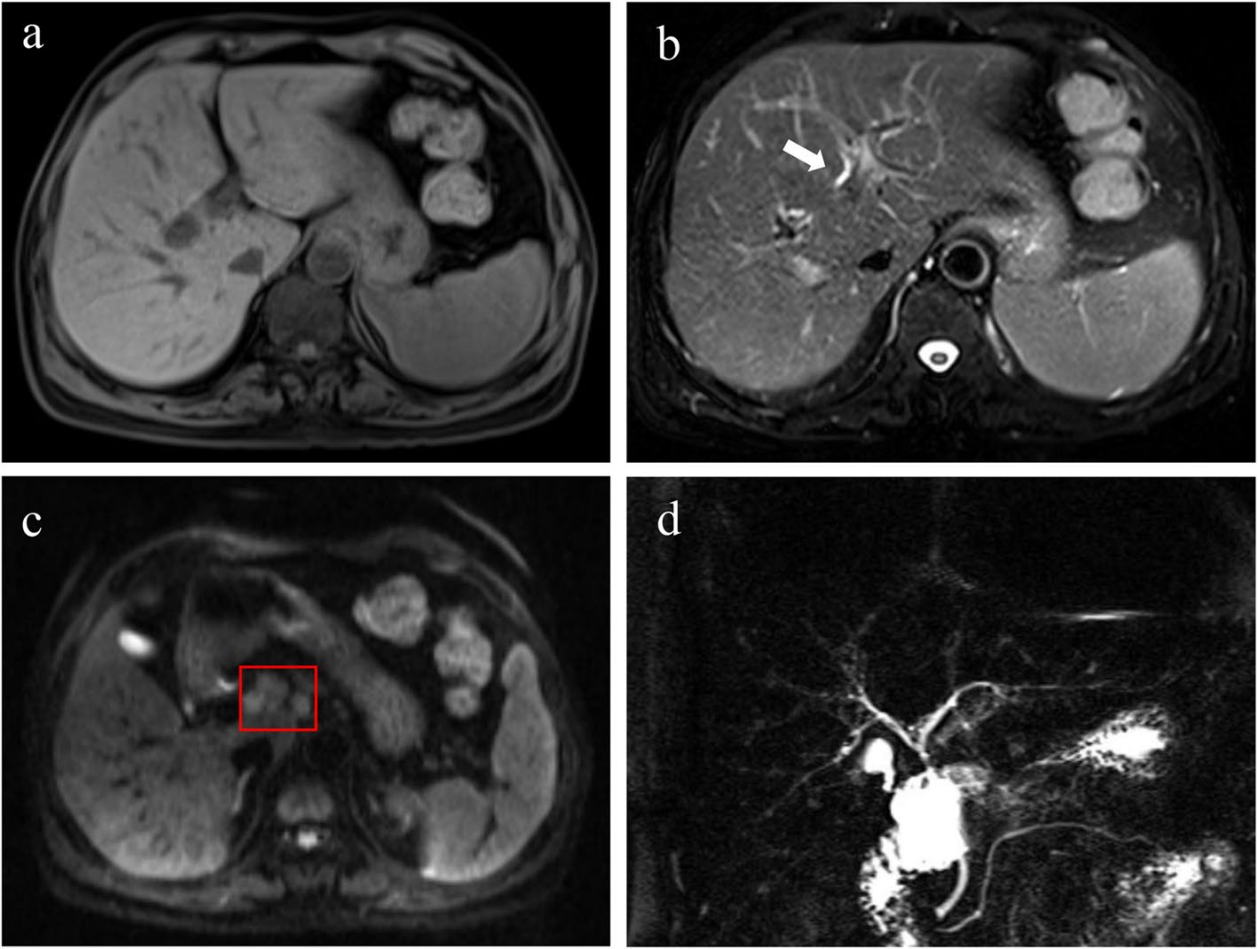
Non-enhanced axial MRI scans in a 52-year-old female with PBC (stage II–III, Ludwig staging system) (**a**–**d**). The T1WI image shows the liver with a regular contour and normal distribution of lobes (**a**). The T2WI image shows periportal hyperintensity (arrows), and mild heterogeneity of liver parenchyma (**b**). The diffusion-weighted image (*b* = 500 s/mm.^2^) shows lymphadenopathy (hepatic hilar, red box) (**c**), and the MRCP image shows irregular morphology, segmental stenosis, and dilatation of the intrahepatic medium-small bile ducts (**d**)

**Fig. 8:**
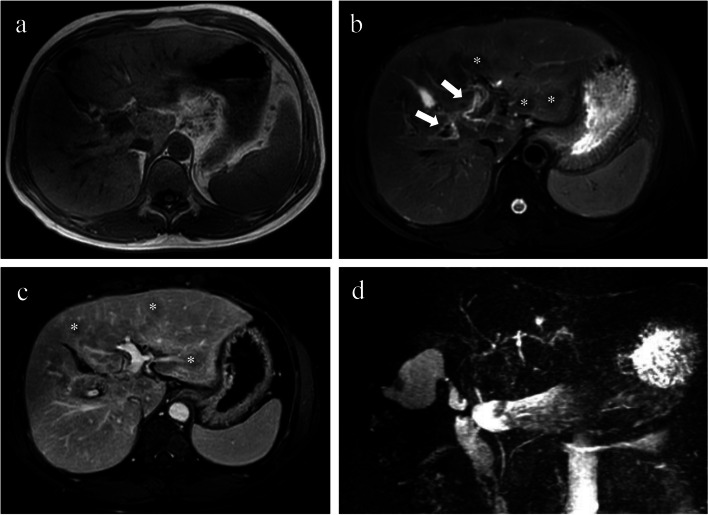
Contrast-enhanced MRI in a 53-year-old female with PBC (stage IV, Ludwig staging system) (**a**–**d**). The T1WI image shows the liver surface nodularity with an irregular contour (**a**). The T2WI image shows periportal hyperintensity (arrows) and periportal halo signs (*) (**b**). The PVP image shows the rounded low signal intensity lesions with no obvious enhancement (*) (**c**), and the MRCP image shows segmental irregular dilatation of the medium to small bile ducts (**d**)

Overall, there are several pathological changes throughout the disease process of PBC that can be captured and monitored by imaging (Figs. 6, 7, and 8). In terms of the CT /MR imaging features, some are relatively unique to PBC, such as liver parenchyma heterogeneity, periportal halo sign, irregular configuration of the small to medium biliary ducts and lymphadenopathy. These features can be better demonstrated on MRI and deserve the attention of radiologists. Thus, they are essential for the early diagnosis of PBC and the timely implementation of clinical management for patients. As the disease progresses, patients with PBC may show signs of liver fibrosis and cirrhosis (i.e. abnormalities in liver morphology, portal hypertension, ascites, and spontaneous peritonitis). These features are more familiar to clinicians and can inform liver transplant decisions. Regarding radiological imaging techniques, CT and MRI have their own advantages and disadvantages for the diagnosis and assessment of PBC, as detailed in Table 2.

**Table 2:** The advantages and disadvantages of CT and MRI for the diagnosis and assessment of primary biliary cholangitis

Techniques	Advantages	Disadvantages	Recommendation
MRI	Multi-sequence, multi-planar, radiation-free, more capable of displaying the characteristic imaging features of PBC, significant advantages for showing bile duct structures	Expensive, long scanning time, more contraindications (patients need to be awake, breath-holding, etc.)	One of the main tools in the diagnosis and differential diagnosis for patients with suspected PBC; the preferred noninvasive tool for the follow-up of patients with early to mild-stage PBC
CT	Inexpensive, fast scanning, high-density resolution, image interpretation is relatively straightforward for clinicians	Radioactive, limited ability to show some of the characteristic features of early PBC, poor performance for bile duct lesions	A diagnostic method for patients with intermediate to advanced PBC to assess the development and progression of portal hypertension, cirrhosis, and related complications

*CT* computed tomography, *MRI* magnetic resonance imaging, *PBC* primary biliary cholangitis

## PBC-AIH variant

Approximately 10–20% of patients with PBC may exhibit clinical symptoms and histological features similar to those of AIH but lack some components of the classical diagnostic criteria in terms of pathology [68, 69]. This condition was formerly called “PBC-AIH overlap syndrome” and was replaced with “PBC-AIH variant”, since not all the patients concurrently present both diseases at the same time [69]. At present, the diagnosis of the PBC-AIH variant is challenging in clinical practice and there are no up-to-date consensus criteria to define it [29]. Liver biopsy is the gold standard for the diagnosis of the PBC-AIH variant and it is also recommended by most authoritative guidelines [1, 20, 69, 70]. Imaging techniques (primarily US) are currently used mainly in the grading of fibrosis in this variant, and their value in the diagnosis of this disease remains to be explored [36, 71, 72].

## Differentiation of PBC from similar diseases

PBC should be differentiated primarily from intrahepatic cholestasis of other causes (e.g. PSC, IgG4-related sclerosing cholangiopathy, sarcoidosis, Langerhans cell histiocytosis (LCH), and secondary biliary cirrhosis) [11] Of these, the distinction between PBC, PSC, and IgG4-related sclerosing cholangiopathy is even more important (Table 3).

**Table 3:** Differential diagnosis between primary biliary cholangitis, primary sclerosing cholangitis and IgG4-related sclerosing cholangitis

Identification points	PBC	PSC	IgG4-related sclerosing cholangitis
Vulnerable populations	Middle-aged woman	Young-aged man	Elderly patients
Possible risk factors	Synergy of multiple factors, including environmental, genetic/epigenetic, and immunological factors	Close relationship with HLA antigens; the intestinal microbiome is key to immune response initiation	Poorly understood
AMA	Presence; highly sensitive and specific for PBC diagnosis; or presence of PBC-specific autoantibodies such as sp100 or gp210 if AMA is negative	Absence	Absence
MRCP features	Segmental small bile duct dilatation, stenosis or poor visualization, mostly involving the intrahepatic secondary bile ducts	Beaded appearance of the intrahepatic and/or extrahepatic bile ducts in the early stages; “pruned-tree” appearance in more advanced stages; the cystic duct, gallbladder, and pancreatic duct may also be affected	Isolated or continuous stricture of the bile duct and upstream dilatation, most commonly affect the intrapancreatic segment of the common bile duct
Concomitant diseases	Rarely combined with inflammatory bowel disease	Often combined with inflammatory bowel disease	Often accompanied by autoimmune pancreatitis, other organs and parts of the body may also be involved

*PBC* primary biliary cholangitis, *PSC* primary sclerosing cholangitis, *HLA* human leukocyte antigen, *AMA* antimitochondrial antibody, *MRCP* magnetic resonance cholangiopancreatography

**Fig. 9:**
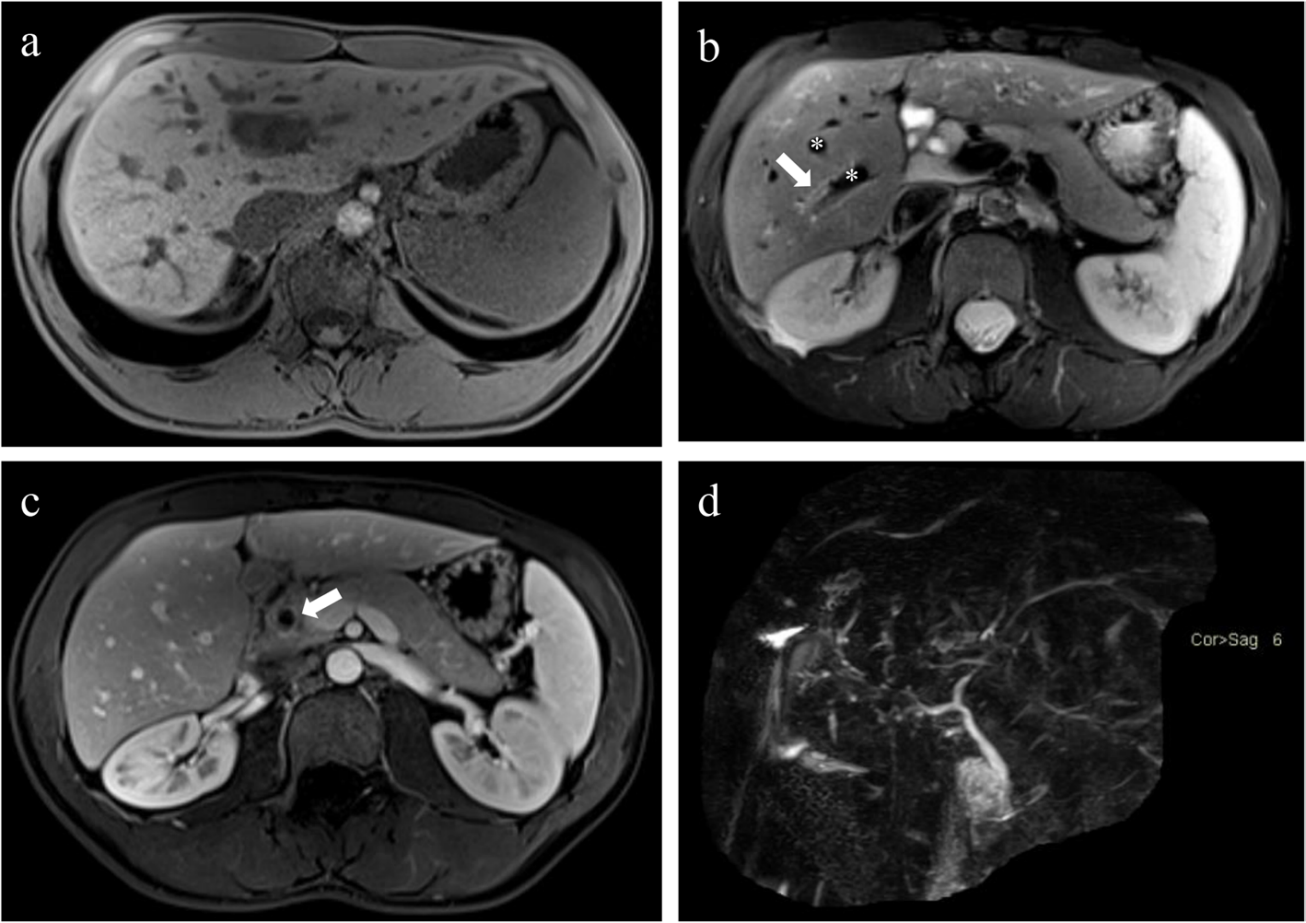
A 22-year-old male PSC patient combined with ulcerative colitis (**a**–**d**); The liver has a regular contour without nodular changes of the surface on T1WI image (**a**). The T2-weighted image shows mild periportal hyperintensity (arrows) and dilated intrahepatic venous ducts (*) (**b**). The PVP image shows the dilatation of the upper common bile duct with heterogeneous enhancement of the duct wall (arrows) (**c**), and the MRCP image shows a branching-tree appearance with saccular dilatations and segmental strictures of the intra- and extrahepatic biliary ducts (**d**)

PSC is also a chronic immune-mediated inflammatory disease involving the bile ducts. However, it differs from PBC in several ways [73, 74]. First, PSC classically occurs in young people aged 30–40 years and is predominantly in males (the ratio of males to females is approximately 2:1). In addition, there is a close relationship between the development of PSC and human leukocyte antigen (HLA) antigens. Furthermore, the intestinal microbiome plays a key role in the initiation of immune responses in the biliary tree. Therefore, there is usually a high probability of diagnosis of irritable bowel disease, including Crohn’s disease and ulcerative colitis, in patients with PSC. On imaging, most PSC cases are observed to involve both the medium- to large-calibre intrahepatic and extrahepatic biliary systems, usually presenting as a branching-tree appearance with saccular dilatations and segmental strictures of the extrahepatic biliary ducts. Moreover, PSC may also involve the cystic duct, gallbladder, and pancreatic duct, which is a point of differentiation from PBC (Fig. 9) [75]. Unlike PBC, patients with PSC have a higher risk of developing cholangiocarcinoma regardless of the degree of fibrosis. About one-thirds of CCAs are detected within 1 year of the diagnosis of PSC. Besides, since 80% of PSC patients may have a combined inflammatory bowel disease, it is advisable to recommend a fusion of CA-199 and colonoscopy, in addition to MRI/MRCP, for the follow-up of PSC patients [76].

**Fig. 10:**
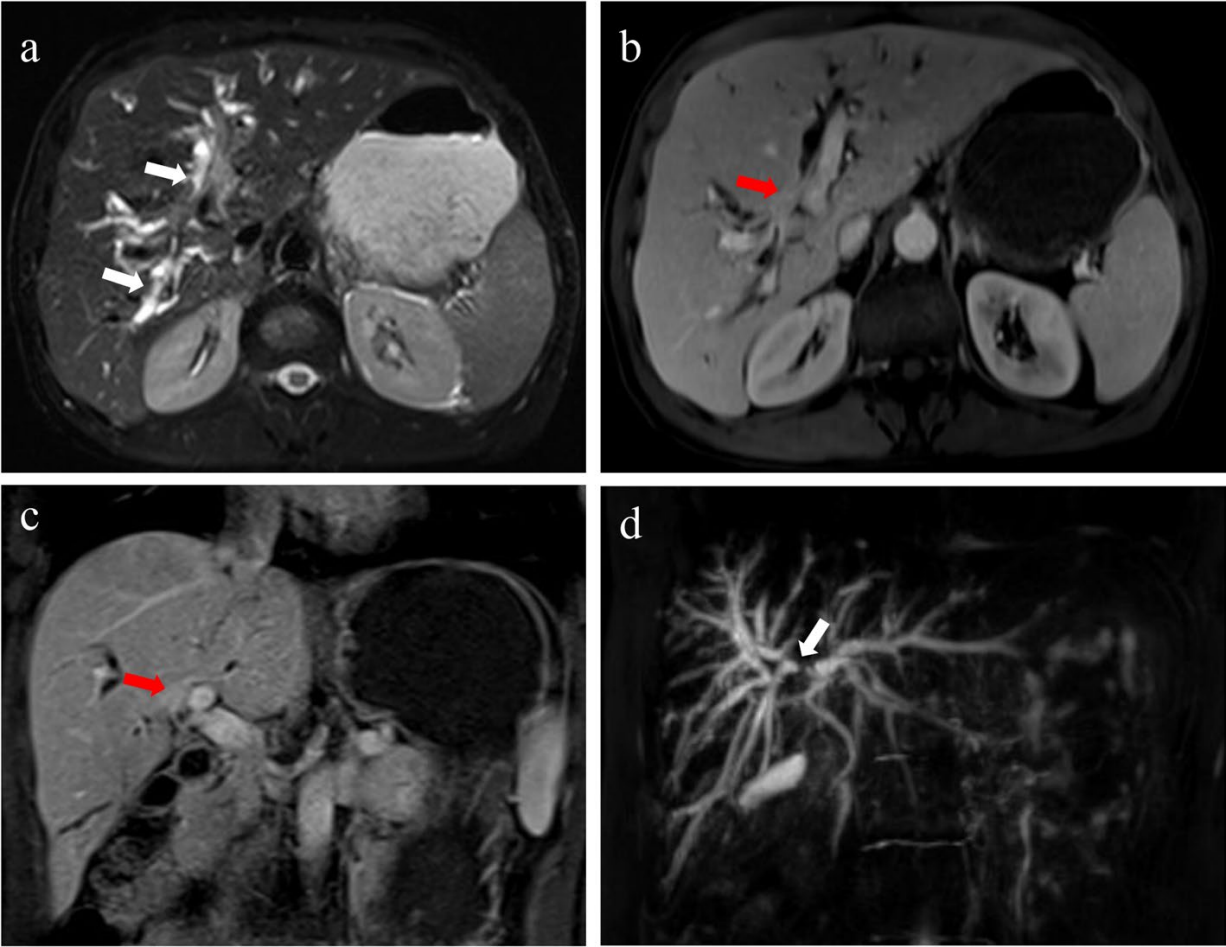
A 51-year-old male with IgG4-related sclerosing cholangitis (**a**–**d**). The T2WI image shows extensive periportal hyperintensity (arrows) (**a**). The axial and coronal images of PVP show the localized thickening of the common hepatic duct wall with moderate enhancement nodules (red arrows), resulting in corresponding bile duct stenosis and truncation (**b**–**c**). The MRCP image shows the localized truncation of the common hepatic duct (arrow) and significant dilatation of intra- and extrahepatic biliary ducts (**d**)

IgG4-related sclerosing cholangitis affects multiple organs and has imaging presentations that are more similar to PSC than PBC [77]. It often affects the distal or intrapancreatic segment of the common bile duct and hepatic bile duct and presents as isolated or continuous stricture or circumferential symmetrical bile duct wall thickening (Fig. 10) [75].

Sarcoidosis (granulomatous cholangitis-induced biliary ductopenia) presents with similar clinical symptoms to PBC. On imaging, intrahepatic granulomas present low signals on both T1WI and T2WI, like the “periportal halo sign” of PBC. However, granulomas usually present mild enhancement, which is different from the “periportal halo sign” [78].

LCH is a rare group of idiopathic disorders caused by the neoplastic proliferation of myeloid cells that infiltrate various tissues and organs [79]. Approximately 10 ~ 15% of cases of LCH involve the bile ducts, so sclerosing cholangitis (often presented as a confined intrahepatic mass, as well as dilated, partially mass-like dilatation of intrahepatic and extrahepatic bile ducts [80–82] can be used to differentiate LCH from PBC.

**Fig. 11:**
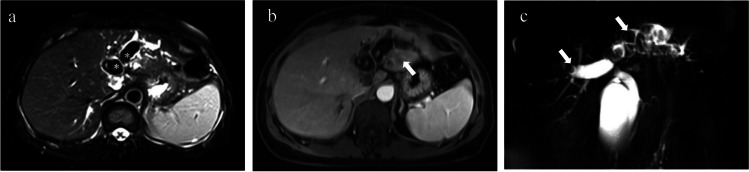
A 50-year-old female with vague right upper abdominal pain with diarrhea. Multiple hypointense nodules (*, gallstone) in the common hepatic duct and the bile ducts of the left lobe of the liver, with corresponding bile duct dilatation, were observed (**a**). Enhancement of the dilated bile duct wall and heterogeneous enhancement (arrow) of the surrounding liver parenchyma can be seen on the PVP image (**b**) and shows the significant dilatation of the common hepatic duct, bilateral hepatic ducts and branches, manifests as a signal void in the MRCP image (arrows) (**c**). Pathological confirmation after surgical resection: intrahepatic cholelithiasis, liver fibrosis (S4) and secondary biliary cirrhosis

**Fig. 12:**
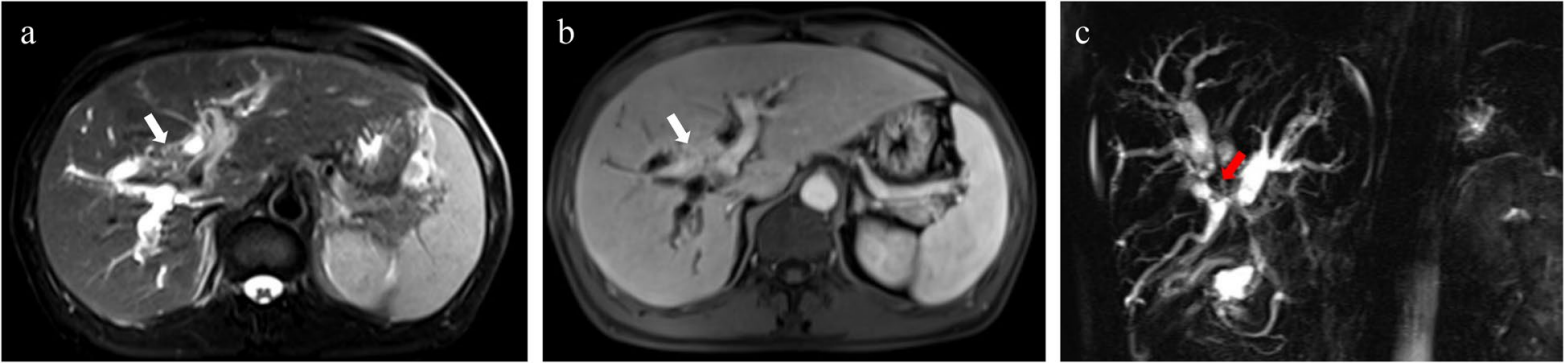
A 54-year-old male with HBV (hepatitis B virus) infection. Moderate hyperintensity nodule with a poorly defined boundary was observed in the T2WI image in the hilar region of the liver (arrow) (**a**); a nodular lesion adjacent to the portal venous confluence and the right branch shows marked enhancement on the PVP image (arrow) (**b**); and the MRCP image shows abrupt interruptions without tapering presented in the hepatic hilar bile ducts (arrow) with vine-like dilatation of the intrahepatic bile ducts (**c**). Pathological confirmation after surgical resection: cholangiocarcinoma of the hilar

Secondary biliary cirrhosis is a form of cirrhosis caused by prolonged obstruction of the large bile duct. Cholelithiasis (Fig. 11) is the most common cause of biliary obstruction and often coexists with cholangitis [83]. Other conditions include carcinoma of the pancreatic head or biliary tree (Fig. 12), postsurgical duct stricture, compression of the bile ducts with enlarged lymph nodes, and biliary atresia (mostly in neonates). Imaging will help to diagnose the level, severity, and cause of biliary obstruction and detect signs of cirrhosis. Calculi can be hyperattenuating to hypoattenuating to bile on CT and manifest as signal voids on T2WI and MRCP. In other settings, stenosis of the lumen of the large bile duct, with or without proximal ductal dilatation, can be seen. Benign stenoses tend to present with smooth symmetrical luminal narrowing, while malignant stenoses often manifest as abrupt transitions without tapering [83].

## Management and treatment monitoring of PBC

As PBC is highly associated with environmental factors, maintaining a healthy lifestyle can help slow the progression of the disease. Patients with PBC should be encouraged to quit smoking, stop drinking alcohol, and maintain an ideal body weight. Moreover, standardized UDCA therapy (13–15 mg/kg/day) should be implemented for all PBC patients. Nevertheless, up to 40% of patients with PBC have an insufficient biochemical response to UDCA and are at high risk of experiencing progression to liver failure or liver transplantation [84]. Hence, the long-term results of second-line drugs such as OCA, fibrates, and budesonide need further verification. It is also necessary to further explore the clinical, imaging and laboratory risk factors to provide individualized care with the available therapeutic agents. Finally, the role of imaging in the assessment of biochemical response after drug treatment for PBC and in the monitoring of disease progression needs to be further explored.

## Conclusion

With the development of imaging technologies, some key pathological alterations in the development and progression of PBC can be captured by CT and MRI. These characteristic intra- and extrahepatic imaging manifestations may reflect the course of the disease and provide information associated with histological grading and altered cellular function. Despite this, the CT or MRI features of PBC are not specific, as many cholestatic liver diseases may present with similar features, and the clinical diagnosis of PBC still requires a combination of imaging, clinical, and laboratory findings.

### Supplementary Information

**Additional file 1: Supplementary Table 1.** The conventional staging systems of primary biliary cholangitis.

## Data Availability

The figure and table are available from the corresponding author, Prof. Bin Song, upon reasonable request.

## Abbreviations

AIH: Autoimmune hepatitis
ALP: Alkaline phosphatase
AMA: Antimitochondrial antibody
APASL: Asia-Pacific Society for the Study of the Liver
APRI: Aspartate aminotransferase to platelet ratio index
AST: Aspartate aminotransferase
BECs: Biliary epithelial cells
CT: Computed tomography
FIB-4: Fibrosis-4
FLIS: Functional liver imaging score
Gd-BOPTA: Gadoxetate disodium
Gd-EOB-DTPA: Gadobenate dimeglumine
GGT: Glutamyl transpeptidase
HLA: Human leukocyte antigen
LCH: Langerhans cell histiocytosis
MR: Magnetic resonance
MRCP: MR cholangiopancreatography
MRE: MR elastography
MRI: MR imaging
OCA: Obeticholic acid
PBC: Primary biliary cholangitis
PDC-E2: E2-subunit of the pyruvate dehydrogenase complex
PSC: Primary sclerosing cholangitis
STE: Sound touch elastography
SWE: Shear wave elastography
T1WI: T1-weighted imaging
T2WI: T2-weighted imaging
UDCA: Ursodeoxycholic acid
USE: Ultrasound elastography
VCTE: Vibration-controlled transient elastography
