# Supplementary material for: CT and MR imaging of primary biliary cholangitis: a pictorial review
Source: Insights Imaging. 2023 Oct 26;14:180. doi: 10.1186/s13244-023-01517-3 (PMC10600092; doi:10.1186/s13244-023-01517-3)
Supplement: Supplementary file 1 — Additional file 1: Supplementary Table 1. The conventional staging systems of primary biliary cholangitis. [file 13244_2023_1517_MOESM1_ESM.docx]

**CT and MR imaging of primary biliary cholangitis：A pictorial review**

**ELECTRONIC SUPPLEMENTARY MATERIAL**

| **Supplementary Table 1: The conventional staging systems of primary biliary cholangitis** | | |
| --- | --- | --- |
| **Staging systems** | **Scheuer staging system** | **Ludwig staging system** |
| Stage I | *The florid duct lesion*: A bile duct is damaged and surrounded by lymphocytes and plasma cells | *Portal Stage*：Portal hepatitis, with little or no periportal inflammation or piecemeal necrosis |
| Stage II | *Ductular proliferation:* All portal tracts are involved; the portal tracts are expanded by young cellular connective tissue in which there are ductules. | *Periportal Stage*: Periportal hepatitis, absence of bridging necrosis and of septal fibrosis |
| Stage III | *Scarring*: Dense fibrous tissue has formed, few ductules and inflammatory cells remain | *Septal Stage*: Fibrous septa (" active septa") or bridging necrosis ("passive septa"), or both |
| Stage IV | *Nodular cirrhosis*: fibrous tissue encroaches extensively on the hepatic parenchyma and nodular regeneration develops | *Cirrhotic Stage*: Fibrous septa and nodular  regeneration |
